# Comprehensive Studies on Detection of Palm Oil Adulteration in Clarified Milk Fat (Ghee)

**DOI:** 10.1155/ijfo/4673218

**Published:** 2025-01-28

**Authors:** Akshay Ramani, Tanmay Hazra, Ayon Tarafdar, Ranjna Sirohi, Anamika Das, Swarnima Dey, Yogesh Kumar

**Affiliations:** ^1^Department of Dairy Chemistry, College of Dairy Science, Amreli, Kamdhenu University, Gandhinagar, Gujarat, India; ^2^Livestock Production and Management Section, ICAR-Indian Veterinary Research Institute, Bareilly, Uttar Pradesh, India; ^3^Sri Karan Narendra Agriculture University, Jobner, Rajasthan, India; ^4^Department of Dairy Chemistry, Sanjay Gandhi Institute of Dairy Technology, Bihar Animal Sciences University, Patna, India; ^5^Department of Food Technology, SRM University, Sonipat, Haryana, India

**Keywords:** adulteration, chromogenic method, GC-FID, ghee, HPLC, milk fat

## Abstract

Heat-clarified milk fat (ghee) is one of the costlier fat-rich dairy products, and palm oil adulteration is a common practice. Limited studies compared traditional physicochemical techniques with advanced chromatographic techniques and chromogenic tests (rapid tests) to ascertain palm oil's presence in ghee. It is a very hard task to ascertain the presence of palm oil in clarified milk fat (ghee) and select the right analytical technique for routine quality control analysis. The present study was designed to validate and compare the physicochemical methods, triglyceride content, phytosterol levels, and rapid chromogenic tests including 2,2-diphenyl-1-picrylhydrazyl (DPPH) or ferric-based assay methods to check the presence of palm oil in clarified milk fat. Physicochemical methods like Reichert–Meissl (RM) value, iodine value (IV), and Butyro refractometer (BR) reading can be used to detect palm oil adulteration below 10%. However, the Kirschner value analysis was able to confirm the presence of palm oil admixture at 5% in clarified milk fat. In chromatographic analytical approaches, triglyceride analysis (*S*-value) was carried out using gas chromatography with flame ionization detection (GC-FID), and plant sterol detection was done by HPLC, which proved to be the robust method for ascertaining the presence of palm oil in milk fat (ghee) at 5%. Chromogenic tests (DPPH and ferric-based assays) provided quick and qualitative assays to detect the presence of palm oil in ghee at a 5% level.

## 1. Introduction

In the current scenario, India stands out as one of the leading milk producers globally with a total production of about 221.06 million tonnes [1]. Ghee, comprising stands as the second most popular dairy product in India, surpassed only by milk. Ghee or clarified milk fat has an integral relation with the Indian diet and subcontinent cuisines [2]. The market size of ghee has been increasing in the last 5 years with a CAGR (compound annual growth rate) of 4.20%[3]. Apart from providing energy, it contains abundant fat-soluble vitamins and bioactive fatty acids: butyric acid (C_4:0_), *cis* and *trans* palmitoleic acid, *α*-linolenic acid (ALA), conjugated linoleic acid (CLA), etc. Scientific studies proved that ghee has numerous health benefits including anticarcinogenic and antidiabetic effects [4–7]; moreover, the application of ghee as traditional medicine is due to its antioxidant and anti-inflammatory properties. Ghee is used in numerous auspicious occasions such as religious ceremonies, and as such, it enjoys a supreme status in India from prehistoric times (Vedic era, 1500 B.C.).

Milk fat adulteration with cheap and unhealthy fats or oils is reported as a widespread issue in the food industry, particularly in developing countries like India [8, 9]. Adulteration of ghee with cheaper vegetable oils or depot body fat poses significant health risks, including heart disease, obesity, and cancer. Addressing this malpractice is crucial not only for consumer safety but also for maintaining consumer trust and ensuring the profitability of the dairy industry.

Adulteration of palm oil in ghee is a widespread issue in some parts of the world including India [10, 11]. Palm oil is a vegetable oil that is widely utilized in the food sector due to its inexpensive nature and being a high-yielding oilseed commodity. It is abundant in saturated and unsaturated fatty acids, with a higher proportion of saturated fats. There are significant differences in the fatty acid and triglyceride compositions between palm oil and milk fat, but their similar physical properties make palm oil an appealing choice for adulteration [12, 13]. Therefore, ascertaining the extent of admixture of ghee with palm oil is always a challenging task [8–11]. Due to the low sensitivity of physicochemical techniques and advancements in adulteration practices, as well as a lack of sophisticated instruments in most of the dairy quality control laboratories, it becomes difficult to ascertain the quality of milk fat or ghee.

Efforts over the past few decades have concentrated on addressing ghee and milk fat adulteration through advancements in physicochemical methods. Sophisticated analytical techniques have been adopted, such as profiling triglycerides and fatty acids using gas chromatography with flame ionization detection (GC-FID) and identifying marker compounds like stigmasterol and *β*-sitosterol via reversed-phase high-performance liquid chromatography (RP-HPLC) ([8, 14–16]. Very recently, Cossignani, Pollini, and Blasi [12] reported that triglyceride analysis was very effective to ascertain the purity of milk fat; later, Van Nguyen et al. [13] analyzed triglyceride of milk fat to detect the presence of nonmilk fat in milk fat. While most of these methods are highly sensitive and effective in detecting adulteration, these techniques cannot be used in routine field conditions due to high operating costs. Ramani et al. [11, 17] proposed rapid tests based on DPPH (2,2-diphenyl-1-picrylhydrazyl) and ferric chloride for instant detection of palm oil addition in ghee. Very recently, the Food Safety and Standards Authority of India (FSSAI) [18] proposed a few tests based on physicochemical analysis, fatty acid analysis, and sterol analysis to ascertain the purity of ghee. However, no rapid method proposed to detect palm oil or other vegetable oil added in ghee or milk fat. To the best of our knowledge, very limited studies compared traditional physicochemical techniques with advanced chromatographic techniques and chromogenic tests (rapid tests) to ascertain palm oil's presence in ghee. The detection of palm oil in ghee has become a challenge to the dairy industry and the selection of the right analytical technique for routine quality control analysis; therefore, this work was designed to validate and compare the level of detection using the physicochemical techniques, advanced chromatographic techniques, and chromogenic test methods that have been developed for detecting palm oil adulteration in ghee.

## 2. Materials and Methods

### 2.1. Sample Collection and Preparation

Cow and buffalo milk were procured from various village cooperative societies affiliated with the Amreli district local dairy farmers. Palm oil (refined) was procured from the local market. Cow and buffalo milk were blended in a 1:1 ratio to prepare mixed ghee samples. The ghee samples were prepared using the direct cream method [19]. This process ensured the production of good quality ghee that was free from any impurities. The ghee samples were used for further study.

The liquid fat sample (40°C) was spiked (mixed) with palm oils at different levels to prepare adulterated milk fat (ghee) samples. The palm oil was added at 5%, 10%, 15%, and 20% v/v in ghee. The ghee without the addition of palm oil was considered as a control sample. All samples were stored at 40°C room temperature for further use.

### 2.2. Ascertaining the Presence of Palm Oil in Clarified Milk Fat (Ghee) Using Physicochemical Constant Analysis

The fat constants, like the Reichert–Meissl (RM) value, iodine value (IV), and Butyro refractometer (BR) reading, were evaluated as per the method described in IS3508:1966 [20]. However, the Kirschner value (KV) of milk fat was evaluated as per the method described by Ghatak and Bandyopadhyay [21].

### 2.3. Chromogenic Assay for Ascertaining the Presence of Palm Oil in Clarified Milk Fat (Ghee)

The two chromogenic methods based on DPPH assay and ferric assay were performed as per the method reported by Ramani et al.[17] and Ramani et al. [11], respectively.

### 2.4. Triglyceride Analysis Using GC-FID Followed by *S*-Value Analysis for Ascertaining the Presence of Palm Oil in Clarified Milk Fat (Ghee)

The triglyceride content in ghee samples was evaluated using GC-FID according to the International Organization for Standardization (ISO) 17678. The analysis was conducted using a GC-FID instrument (Agilent 7890B). In an Erlenmeyer flask, 1 g of sodium sulfate was used to filter 50 g of the melted sample. In accordance with ISO 17678: 2010 guidelines, a 1% filtered milk fat solution in n-hexane was prepared and injected (1 *μ*L) into GC-FID. Using certified reference standards, 16 peaks were identified and *S*-value was calculated using the formula indicated in [22]).

*S*-values are calculated by inserting the estimated % mass of the relevant triglyceride fractions into standardized equations (Table S1). The five calculated *S*-values are then compared with their respective limits specified as standards (Table S1). The results are interpreted based on whether the *S*-values meet the specified limits. If all five *S*-values for a test sample fall within the specified limits, the sample is deemed pure. However, if one or more *S*-values fall outside of these limits, the sample is considered adulterated. While individual *S*-values (i.e., S1, S2, S3, and S4) can be more sensitive to certain foreign fats than the overall *S*-value (*S* total), a positive result in just one *S*-value is not sufficient to determine the type of foreign fat present.

### 2.5. RP-HPLC-Based Approach for Sterol Analysis for Ascertaining the Presence of Palm Oil in Clarified Milk Fat (Ghee)

One gram of ghee was weighed in a round bottom flask, and 25 mL of a 5% methanolic potassium hydroxide (KOH) solution was added. The flask was placed in a water bath set at a constant 90°C for around 50 min, with periodic shaking, and was attached to a condenser. After saponification was complete, 5 mL of water and 15 mL of hexane were added. This mixture was then subjected to centrifugation for 5 min at 3000 rpm. The top hexane layer was recovered and dried to form Unsaponifiable matter. For RP-HPLC, a 0.22 *μ* syringe filter was used to filter the sample dissolved in chloroform.

The quantification of plant sterol was conducted using the method reported by Rani et al. [14]. An HPLC (Agilent 1260) system with a UV detector was used to identify *β*-sitosterol and stigmasterol in the ghee. A 20 *μ*L sample was injected into the HPLC column (reversed Phase C-18, 4.6 × 250 mm ID, 5 *μ*m, 120 Å particle size, Dionex), maintained at 30°C in a temperature-controlled column oven, for the separation of sterols. Chromatography was performed with a linear solvent gradient (acetonitrile: isopropanol; 9:1, v/v) at a flow rate of 1.5 mL/min over 30 min. Sterols were detected using a UV detector set at 205 nm.

### 2.6. Statistical Analysis

In this study, data were analyzed using IBM SPSS, Version 25, and the findings were reported as mean ± standard deviation. One-way analysis of variance (ANOVA) at a 5% level of significance and Tukey's honestly significant difference (HSD) as a post hoc test were also applied to the data.

## 3. Results and Discussion

The ghee samples spiked with palm oil were subjected to analysis for different parameters and the observations are described below.

### 3.1. BR Reading for Detecting Ghee Samples Spiked With Palm Oil

BR reading helps to determine the level of saturation/unsaturation of fatty acids in particular oils and fats. The BR reading increases as unsaturation increases, while the BR reading decreases as unsaturation decreases [15]. In this study, the BR reading of pure ghee was 42.96. Adulteration with palm oil increased BR readings proportionally (Table 1). From Table 1, it was observed that BR readings were directly proportional to the level of added palm oil, indicating a significant (*p* < 0.05) difference in BR reading for ghee adulterated with palm oil at 10% or higher. Gandhi and Lal [23] reported that BR readings were unable to detect adulteration in ghee at a lower level of 5%. In a distinct study, El-Nabawy, Awad, and Ibrahim [24] reported that the difference of BR reading for pure and palm oil adulterated milk fat did not vary widely; therefore, BR reading was not an effective parameter for detecting palm oil adulteration in ghee or milk fat.

### 3.2. RM Value for Detecting Ghee Samples Spiked With Palm Oil

The RM value is an important parameter that indicates the presence of short-chain, steam-volatile, water-soluble fatty acids, indicating mainly butyric acid (C_4:0_) and caproic acid (C_6:0_). It was observed that the RM value for pure ghee was observed to be 31.38; however, for palm oil added in ghee, the RM value decreased accordingly (Table 1). The RM value was significantly reduced due to the absence of C_4:0_ or C_6:0_ fatty acids in palm oil. It was also observed that ghee adulterated with palm oil even at 5%, the RM value was significantly (*p* < 0.05) reduced. In previous studies, researchers faced challenges in determining the presence of vegetable oil in milk fat or ghee when it was at lower levels [15, 24]. The present study also confirmed that adulteration of palm oil in ghee reduces the RM value of ghee. The present study also confirmed that RM value analysis alone was unable to determine the presence of palm oil in ghee.

### 3.3. KV Analysis for Detecting Ghee Samples Spiked With Palm Oil

The KV is an important parameter that indicates the presence of short-chain steam-volatile water-insoluble fatty acids with silver salt (AgNO_3_). This value is mainly dependent on butyric acid (C_4:0_). The KV of pure ghee was found to be 25.53, which decreased with the addition of palm oil (Table 1). Admixed ghee with palm oil had a significantly lower KV than pure ghee. The KV of adulterated ghee samples decreased with the increase in the level of palm oil addition (Table 1). The KV showed an inversely proportional trend with the level of added palm oil. The data in Table 1 shows substantial differences (*p* < 0.05) between pure ghee and ghee adulterated with even a level of 5%. Analysis of KV proved an effective method to detect palm oil adulteration in ghee even at a 5% level. Butyric acid (C_4:0_) is present in milk fat, making the KV of ghee higher than that of palm oil, and the value decreases with an increase in the quantity of palm oil.

### 3.4. IV for Detecting Ghee Samples Spiked With Palm Oil

In this study, the IV of pure ghee was found to be 29.21. Adulteration with palm oil increased IV proportionally (Table 1). The present investigation also revealed that significant (*p* < 0.05) increase in IV than pure ghee when palm oil was added at 10% or higher level. The IV measures the unsaturation of fat by calculating the grams of iodine absorbed per 100 g of fat. The IV varies depending on the type and proportion of unsaturated fatty acids in the fat. Palm oil has quite a higher IV than ghee or milk fat [25]. It could be assumed that adulteration of palm oil in ghee increased the unsaturated fatty acids that directly influenced the IV of ghee. The increase in unsaturated fatty acids caused by palm oil leads to a proportionate increase in IV. Gandhi, Kumar, and Lal [25] reported a similar result for the detection of palm oil added in ghee. The present study confirmed that IV analysis was unable to ascertain the purity of ghee at a lower level (at 5%) of adulteration of palm oil in ghee.

### 3.5. Chromogenic Tests to Detect Ghee Samples Spiked With Palm Oil

#### 3.5.1. DPPH-Based Chromogenic Test

**Figure 1:**
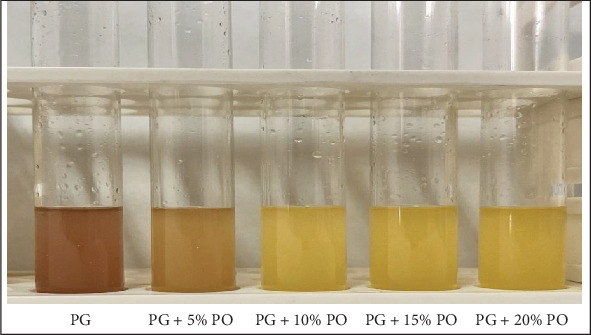
Reaction between ghee samples and DPPH dye after 5 min of conductance of test.

A rapid chromogenic test to ascertain the purity of ghee is the need of time; therefore, in this present study, a rapid DPPH-based chromogenic test was employed to determine the purity of ghee. It was observed that adulterated ghee turned yellow, but pure ghee remained violet after completion of the test (Figure 1). This indicated that the chromogenic test was able to detect even a 5% level of adulteration of palm oil in ghee, since DPPH is a chemically stable molecule that accepts an electron or a free radical species, thus causing a color change from violet to a pale yellow colored complex due to the presence of the picryl group [26]. According to Mba, Dumont, and Ngadi [27], palm oil has been an excellent source of natural antioxidants such as vitamin E, carotene, phytosterols, phenolic compounds, and phospholipids. Synthetic antioxidants are often mixed with palm oil to retard oxidation. It could be assumed that ghee adulterated with palm oil has increased antioxidant compounds, thus donating free electrons to DPPH. As a result, the color changed, whereas in pure ghee, due to the insufficient antioxidants, the color remains violet (Figure 1). Thus, it was envisaged that the DPPH-based rapid chromogenic test could detect palm oil in ghee up to a concentration of 5% or more. This observation aligns with the findings of Ramani et al. [17]. A similar study conducted on milk successfully detected palm oil in concentrations of 5% or more using fat extraction techniques [28].

#### 3.5.2. Ferric Assay–Based Test Chromogenic Test

This rapid chromogenic assay was earlier used by Ramani et al. [11] to detect the added palm oil in ghee. In this test, the color changed from light green to Prussian blue due to substances with a reduction potential that can react with potassium ferric cyanide. This reaction then proceeds with ferric chloride to create a deep blue ferric ferrous complex, with a peak absorption at 700 nm [29]. Neo et al. [30] proposed the presence of natural antioxidants that possess reducing capacities. These antioxidants include carotenoids such as *α*, *β*, and ɤ carotenes, as well as vitamin E in the form of tocopherols and tocotrienols. Additionally, sterols like *β*-sitosterol, stigmasterol, and campesterol, along with phospholipids, glycolipids, and squalene, are identified as potent water-soluble antioxidants.

**Figure 2:**
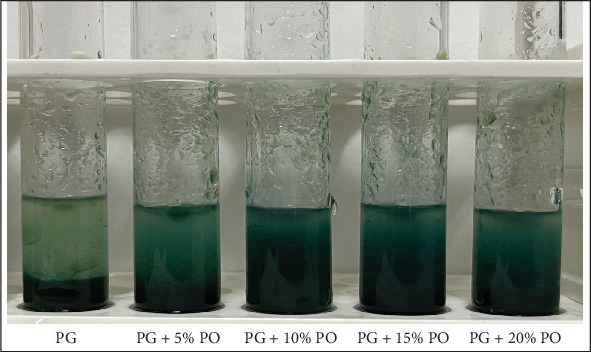
Reaction between ghee samples, ferric chloride, and potassium ferricyanide after 5 min of conductance of test.

The present study clearly confirmed that the color of spiked ghee (5%, 10%, 15%, and 20%) samples changed to Prussian blue color (Figure 2). The addition of palm oil to ghee leads to an increase in the amount of natural antioxidants, leading to a noticeable change in the chromogenic solution's color to a rich blue tint. Although the pure ghee sample did not exhibit any color change, this can be attributed to the insufficient amount of antioxidants present in pure ghee. Hence, the ferric assay-based chromogenic test is able to detect ghee adulterated with palm oil at a 5% level.

The present study confirmed that both rapid chromogenic tests are able to detect 5% palm oil in ghee; therefore, these two techniques could be used to screen out adulterated ghee samples in field conditions as well as in the rural dairy industry where there is a lack of sophisticated analytical instrument [31]. Rapid chromogenic methods for ascertaining the purity of ghee are the need of the hour, and therefore, these two tests can be utilized to detect palm oil in ghee, especially in field condition.

### 3.6. Chromatography-Based Analytical Approach to Detect Ghee Samples Spiked With Palm Oil

#### 3.6.1. Triglyceride Analysis Using GC-FID Followed by *S*-Value Analysis

The ISO 17678: 2010 method for analyzing triglycerides is an efficient technique to determine the purity of milk fat. The *S*-values of milk fat are calculated based on the percentages of different triglyceride fractions present in it. Since fatty acids, which are integral parts of triglycerides, determine the number of carbon atoms in triglyceride molecules, the chain length of these fatty acids affects the triglyceride composition. Therefore, the percentages of short-chain (C_4:0_ to C_14:0_), medium-chain (C_16:0_ and C_16:1_), and long-chain (C_18:0_, C_18:1_, and C_18:2_) fatty acids in milk fat influence the triglyceride fractions and, consequently, the *S*-values [32]. The method established a characteristic triglyceride profile for pure milk fat, featuring 16 peaks with even carbon numbers ranging from 24 to 54. Most plant oils and animal body fats are primarily composed of long-chain fatty acids, followed by medium-chain fatty acids, with little to no short-chain fatty acids. Changes in the fatty acid composition of milk fat lead to corresponding changes in its triglyceride profile [32].

To account for this, ISO 17678 has set “S” limits based on five regression equations calculated from the triglyceride profile of pure milk fat, encompassing 14 foreign fats; this includes 11 vegetable oils and 3 animal body fats (Table S1). Limits of *S*-values for pure ghee and adulterated ghee samples specific limits are described in Table S2. Triglyceride analysis, followed by the calculation of S-limits for pure ghee and adulterated ghee (milk fat) samples, is presented in Table 2. Chromatograms of different samples analyzed using GC-FID are shown in Figures S1 and S2. The method can detect the presence of palm oil in ghee or milk fat, and it is effective in identifying palm oil adulteration at or above 5%. So, this method could be adopted by any regulatory body to ascertain the quality of ghee.

#### 3.6.2. RP-HPLC-Based Approach for Sterol Analysis

The plant sterols, stigmasterol, and *β*-sitosterol, which are principal sterols, can be commonly used as markers to detect adulteration of vegetable oils in milk fat [14]. The absence of plant sterols in pure ghee is indicated in Table 3, whereas adulteration of pure ghee with palm oil at 5%, 10%, 15%, and 20% levels resulted in an increase in the concentration of stigmasterol and *β*-sitosterol. Based on the observed trend, it could be concluded that *β*-sitosterol served as a more effective marker in comparison to stigmasterol. Nevertheless, the inclusion of *β*-sitosterol in ghee has the potential to yield erroneous positive outcomes. Thus, using stigmasterol as an additional marker can improve the accuracy of the results [15]. The absence of plant sterols in pure ghee is indicated in Table 3 (Figure S3), whereas adulteration of pure ghee with palm oil resulted in an increase in the concentration of stigmasterol and *β*-sitosterol in Table 3 (Figure S4). Rani et al. [14] reported that the HPLC-based method was very efficient to detect other oils including coconut, soya bean, sunflower, and peanut oil. However, this is a very time-consuming method.

## 4. Conclusion

The present study represented the different physicochemical, chromatographic, and rapid chromogenic methods and their level of detection for ascertaining the presence of palm oil in ghee. It was observed that both BR reading and IV analysis were not as efficient in detecting palm oil adulteration in ghee at lower level. RM value analysis alone was not able to draw the conclusion regarding the purity of ghee. However, KV analysis could be an effective parameter to detect adulteration of palm oil in ghee. DPPH and ferric chloride-based chromogenic tests were very effective to detect the presence of palm oil in milk fat or ghee rapidly; thus, these tests could be used in field conditions. The use of triglyceride analysis (*S*-value) and plant sterol detection offered a comprehensive approach to detect palm oil adulteration in ghee at 5% levels. These methods collectively can be used to authenticate the quality of ghee samples in the dairy industry, ensuring product purity and consumer safety.

## Figures and Tables

**Table 1:** Physicochemical analysis of pure ghee and adulterated ghee samples.

**Sample**	**BR reading**	**RM values**	**Kirschner value**	**Iodine value**
PG	42.96 ± 0.31^d^	31.38 ± 0.73^a^	25.29 ± 0.37^a^	29.21 ± 0.52^d^
PG + 5% PO	43.18 ± 0.02^d^	30.42 ± 1.01^b^	24.35 ± 0.09^b^	29.71 ± 0.20^d^
PG + 10% PO	43.75 ± 0.13^c^	28.28 ± 0.30^c^	23.4 ± 0.25^c^	31.6 ± 0.37^c^
PG + 15% PO	44.11 ± 0.39^b^	26.74 ± 0.67^d^	22.06 ± 0.53^d^	32.57 ± 0.01^b^
PG + 20% PO	44.46 ± 0.03^a^	23.28 ± 1.12^e^	20.8 ± 0.36^e^	33.14 ± 0.43^a^

*Note:* Data are presented as mean ± SD, and data with different letters within a column show significant (*p* < 0.05) differences from each other.

Abbreviations: PG, pure ghee; PO, palm oil.

**Table 2:** Standardized (S)-limits of pure ghee and adulterated ghee samples.

**S-limits for pure cow milk fats as per ISO standard**		**PG**	**PG + 5% PO**	**PG + 10% PO**	**PG + 15% PO**	**PG + 20% PO**
**Equations**	**Limits**					
S2 (sunflower, soya bean, rape seed, olive, linseed, maize germ, wheat germ, cotton seed, and fish oil)	98.05–101.95	99.40 ± 0.11	99.16 ± 0.32	98.95 ± 0.18	99.15 ± 0.24	99.28 ± 0.14
S3 (coconut and palm kernel fat)	99.42–100.58	99.97 ± 0.45	100.17 ± 0.25	100.43 ± 0.22	100.71 ± 0.37	101.02 ± 0.88
S4 (palm oil and beef tallow detection)	95.90–104.10	100.34 ± 0.21	95.37 ± 0.19	90.99 ± 0.17	86.70 ± 0.05	81.54 ± 0.54
S5 (lard)	97.96–102.04	100.10 ± 0.17	101.66 ± 0.15	102.98 ± 0.56	104.07 ± 0.25	105.99 ± 0.19
S total (total general formula)	95.68–104.32	99.30 ± 0.23	94.28 ± 0.22	89.88 ± 0.28	85.66 ± 0.22	80.24 ± 0.43

*Note:* All outcomes were mean of replicates.

**Table 3:** Plant sterol detection using HPLC-PDA.

**Sample**	**Plant sterol**	
	**Stigmasterol (mg/100 g)**	***β*-Sitosterol (mg/100 g)**
PG	ND	ND
PG + 5% PO	8.24 ± 0.25	12.55 ± 0.89
PG + 10% PO	14.82 ± 1.02	23.26 ± 0.56
PG + 15% PO	27.22 ± 0.93	43.13 ± 1.23
PG + 20% PO	40.54 ± 1.59	78.22 ± 1.87

*Note:* All outcomes were mean of replicates.

Abbreviation: ND, not detected.

## Data Availability

Data will be made available on request.

## Supporting Information

Supporting Information Additional supporting information can be found online in the Supporting Information section. Figure S1: GC-FID chromatographs of pure ghee showing different triglyceride contents which have been used for *S*-value calculation. Figure S2: GC-FID chromatographs of pure ghee adulterated with palm oil showing different triglyceride contents which have been used for *S*-value calculation. Figure S3: HPLC chromatographs of pure ghee. Figure S4: HPLC chromatographs of ghee adulterated with palm oil showing the additional peaks of stigmasterol and beta-sitosterol. Table S1: Equation for calculating the *S*-value of pure ghee and adulterated ghee samples. Table S2: Specification on limits of *S*-values for pure ghee and adulterated ghee samples.

## References

[1] NDDB Report (2023). Milk production in India. https://www.nddb.coop/information/stats/milkprodindia.

[2] Battula S. N., Naik N. L., Sharma R., Mann B. (2020). Ghee, anhydrous milk fat and butteroil. *Dairy Fat Products and Functionality: Fundamental Science and Technology*.

[3] Global ghee market report and forecast (2023). https://www.expertmarketresearch.com/reports/ghee-market.

[4] Bali S., Utaal M. S. (2019). Ghee: the much maligned cooking medium, now slowly reclaiming its therapeutic reputation. *International Journal*.

[5] Basak S., Duttaroy A. K. (2020). Conjugated linoleic acid and its beneficial effects in obesity, cardiovascular disease, and cancer. *Nutrients*.

[6] Gandhi K., Sharma R., Seth R., Ramani A., Mann B. (2023). Mineral oil detection in ghee using attenuated total reflectance-Fourier transform infrared spectroscopy (ATR-FTIR) in conjunction with chemometrics. *Food and Humanity*.

[7] Sonvanshi V., Gandhi K., Ramani A., Sharma R., Seth R. (2024). ATR-FTIR coupled with chemometric techniques to detect vanaspati ghee (hydrogenated vegetable oil) adulteration in milk fat. *Results in Chemistry*.

[8] Aparnathi K. D., Sharma S., Antony B., Mehta B. M. (2019). Development of method for detection and quantification of foreign oils and fats in ghee (heat clarified milk fat) using FT NIR spectroscopy couple with chemometric. *Indian Journal of Dairy Science*.

[9] Hazra T., Sudheendra C. V. K., Ahuja K. K., Sindhav R. G., Ramani V. M. (2020). A study on change of fatty acids profile in ghee adulterated with palm oil. *Indian Journal of Dairy Science*.

[10] Kauser H., Shilpashree B. G., Ashwini A. (2023). Innovative techniques to assess adulteration in ghee. *Current Perspectives in Agriculture and Food Science*.

[11] Ramani A., Hazra T., Parmar M. P., Sindhav R. G., Ramani V. M. (2019). A simple rapid technique for detection of palm oil in ghee. *Indian Journal of Dairy Science*.

[12] Cossignani L., Pollini L., Blasi F. (2019). Invited review: authentication of milk by direct and indirect analysis of triacylglycerol molecular species. *Journal of Dairy Science*.

[13] Van Nguyen A., Thi Ngoc Vu A., Deineka V. I., Deineka L. A., Kien Thi Linh Da T. (2023). Novel approach for determination of milk fat adulteration with non-milk fat by RP-HPLC. *Journal of Food Composition and Analysis*.

[14] Rani A., Sharma V., Arora S., Ghai D. L. (2015). Comparison of rapid reversed phase high-performance liquid chromatography (RP-HPLC) method with rapid reversed phase thin layer chromatography method for detecting vegetable oils in ghee (clarified milk fat). *International Journal of Food Properties*.

[15] Shinde D., Darji H., Chawla R. (2020). Application of physico-chemical and chromatographic techniques for detection of adulteration in ghee (milk fat). *Indian Journal of Dairy Science*.

[16] Upadhyay A. K. N., Padghan P. V., Gandhi K., Lal D., Sharma V. (2015). Detection of vegetable oil and animal depot fat adulteration in anhydrous milk fat (ghee) using fatty acid composition. *MOJ Food Processing & Technology*.

[17] Ramani A., Hazra T., Sudheendra C. V. K., Hariyani A. S., Prasad S., Ramani V. M. (2018). Comparative appraisal of ghee and palm oil adulterated ghee on the basis of chromogenic test. *International Journal of Current Microbiology and Applied Sciences*.

[18] FSSAI (2021). *Food Safety and Standards (Food Products Standards and Food Additives) Regulations, 2011. Amendment in Force From 27th December, 2021*.

[19] De S. (1980). *Outlines of Dairy Technology*.

[20] IS3508:1966 (2018). *Methods of Sampling and Test for Ghee (Butter Fat)*.

[21] Ghatak P. K., Bandyopadhyay A. K. (2007). *Practical Dairy Chemistry*.

[22] ISO17678/IDF202:2010(E) (2010). Determination of milk fat purity by gas chromatographic analysis of triglycerides.

[23] Gandhi K., Lal D. (2017). Butyro-Refractometer (B.R.) reading linked with solvent fractionation technique as an aid to detect adulteration of palm olein and sheep body fat in ghee. *Indian Journal of Natural Products and Resources*.

[24] El-Nabawy M., Awad S., Ibrahim A. (2023). Validation of the methods for the non-milk fat detection in artificially adulterated milk with palm oil. *Food Analytical Methods*.

[25] Gandhi K., Kumar A., Lal D. (2015). Iodine value integrated with solvent fractionation technique as a tool for detecting palm olein and sheep body fat adulteration in ghee (clarified milk fat). *Indian Journal of Dairy Science*.

[26] Joshi M. (2015). *Standardization of a Method to Distinguish Cotton Tract Ghee From the Ghee Adulterated With Cottonseed Oil*.

[27] Mba O. I., Dumont M.-J., Ngadi M. (2015). Palm oil: processing, characterization and utilization in the food industry–a review. *Food Bioscience*.

[28] Hazra T., Ramani A., Hariyani A. S., Sudheendra C. H. V. K., Ajuha K., Ramani V. M. (2018). A simple rapid chromogenic test for detection of palm oil adulteration in milk. *International Journal of Chemical Studies*.

[29] Jayanthi P., Lalitha P. (2011). Determination of the in vitro reducing power of the aqueous extract of Eichhornia crassipes (Mart.) Solms. *Journal of Pharmacy Research*.

[30] Neo Y. P., Ariffin A., Tan C. P., Tan Y. A. (2008). Determination of oil palm fruit phenolic compounds and their antioxidant activities using spectrophotometric methods. *International Journal of Food Science & Technology*.

[31] Akshay R., Sen C., Hazra T., Sindhav R. G. (2023). Novel ghee adulteration detection methods. *Ghee*.

[32] Aparnathi K. D., Patel A., Mehta B. M., Patel D., Prajapati J. B. (2024). Gas chromatographic analysis of triglycerides-the reference method for testing purity of milk fat and perspectives on its use in india: a review: GC analysis of triglycerides for testing purity of milk fat. *Indian Journal of Dairy Science*.

