# Supplementary material for: Comprehensive Studies on Detection of Palm Oil Adulteration in Clarified Milk Fat (Ghee)
Source: Int J Food Sci. 2025 Jan 28;2025:4673218. doi: 10.1155/ijfo/4673218 (PMC11824851; doi:10.1155/ijfo/4673218)
Supplement: Supporting Information — Additional supporting information can be found online in the Supporting Information section. Figure S1: GC-FID chromatographs of pure ghee showing different triglyceride contents which have been used for S-value calculation. Figure S2: GC-FID chromatographs of pure ghee adulterated with palm oil showing different triglyceride contents which have been used for S-value calculation. Figure S3: HPLC chromatographs of pure ghee. Figure S4: HPLC chromatographs of ghee adulterated with palm oil showing the additional peaks of stigmasterol and beta-sitosterol. Table S1: Equation for calculating the S-value of pure ghee and adulterated ghee samples. Table S2: Specification on limits of S-values for pure ghee and adulterated ghee samples. [file 4673218.f1.docx]

**Supplementary Figures**

**Figure S1** GC-FID chromatographs of pure ghee

**Figure S2**GC-FID chromatographs of pure gheeadulterated with palm oil

**Figure S3**HPLC chromatographs of pure ghee

**Figure S4** HPLC chromatographs of ghee adulterated with palm oil

**
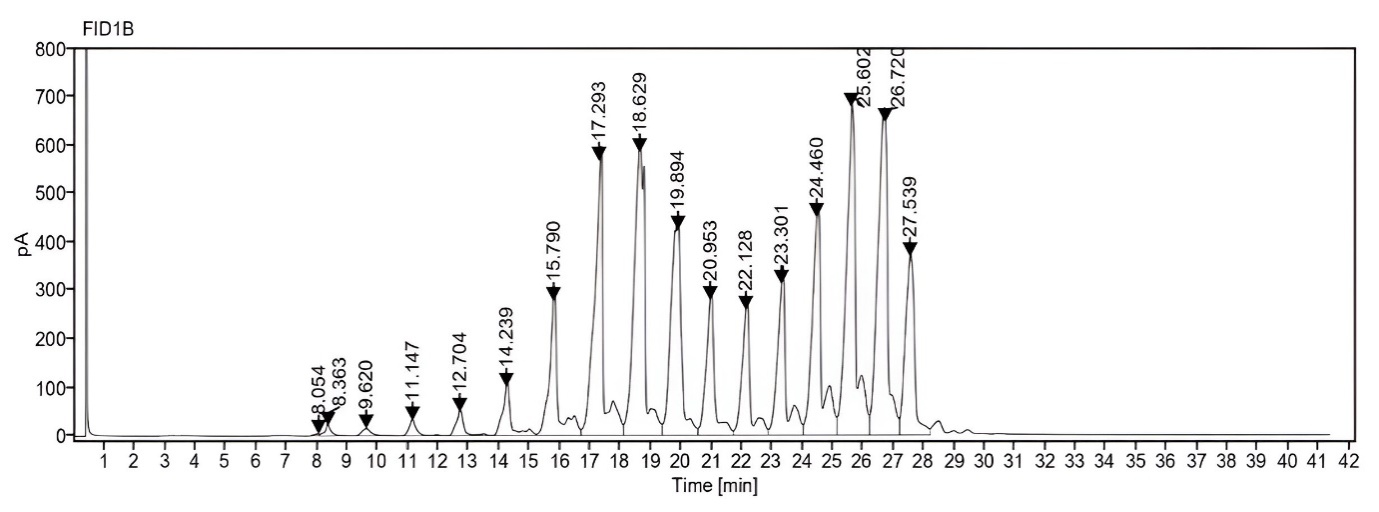
**

**Figure S1**

**
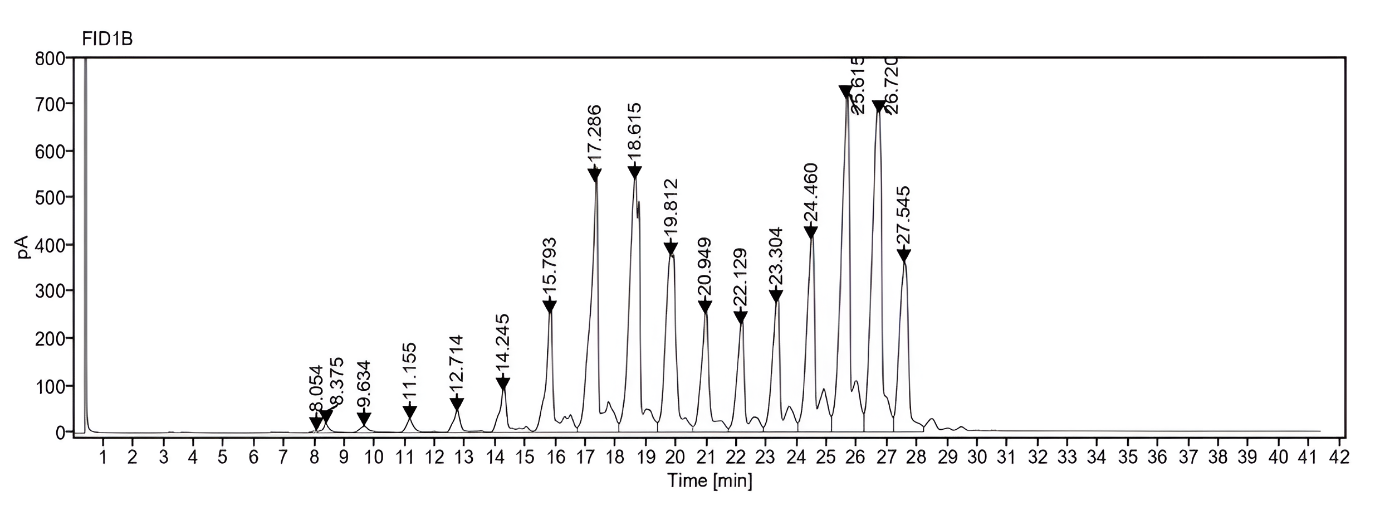
**

**Figure S2**

**
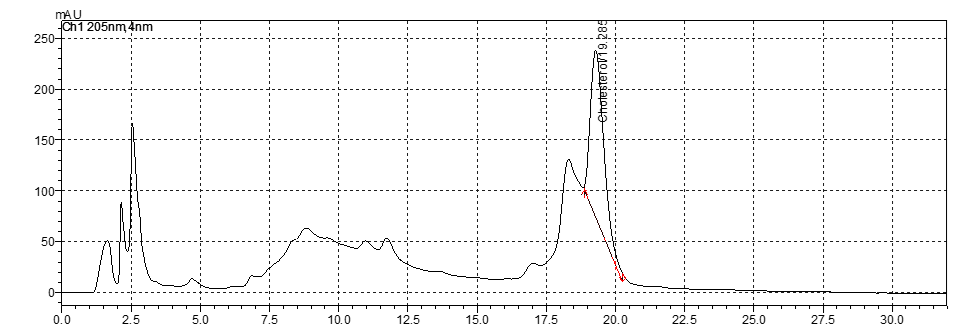
**

Cholesterol

**Figure S3**

**
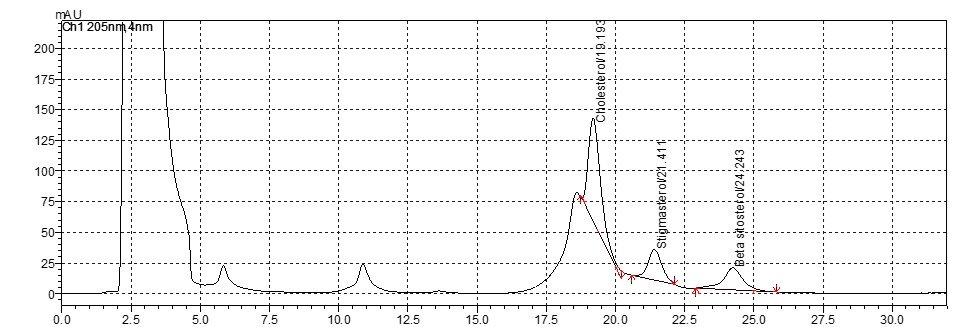
**

Betasitosterol

Cholesterol

Stigmasterol

**Figure S4**

**Supplementary Table**

**Table S1** Equation for calculate the S-value of pure ghee and adulterated ghee samples.

**Table S2** Specification on limits of S-values for pure ghee and adulterated ghee samples.

**Triglyceride analysis using GC-FID followed by S-value analysis**

The ISO 17678:2010 method for analyzing triglycerides is an efficient technique to determine the purity of milk fat. The S-values of milk fat are calculated based on the percentages of different triglyceride fractions present in it. Since fatty acids, which are integral parts of triglycerides, determine the number of carbon atoms in triglyceride molecules, the chain length of these fatty acids affects the triglyceride composition. Therefore, the percentages of short-chain (C_4:0_ to C_14:0_), medium-chain (C_16:0_ and C_16:1_), and long-chain (C_18:0_, C_18:1_, and C_18:2_) fatty acids in milk fat influence the triglyceride fractions and, consequently, the S-values (Aparnathi*et al*., 2024). The method established a characteristic triglyceride profile for pure milk fat, featuring 16 peaks with even carbon numbers ranging from 24 to 54. Most plant oils and animal body fats are primarily composed of long-chain fatty acids, followed by medium-chain fatty acids, with little to no short-chain fatty acids. Changes in the fatty acid composition of milk fat lead to corresponding changes in its triglyceride profile (Aparnathi*et al.*, 2024).

To account for this, ISO 17678 has set ‘S’ limits based on five regression equations calculated from the triglyceride profile of pure milk fat, encompassing 14 foreign fats, this includes 11 vegetable oils and 3 animal body fats (Table S1). Limits of S-values for pure ghee and adulterated ghee samples specific limits describe in Table S2. Triglyceride analysis, followed by the calculation of S-limits for pure ghee and adulterated ghee (milk fat) samples, is presented in Table 2. Chromatograms of different samples analyzed using GC-FID are shown in Figures S1–S5. The method can detect the presence of palm oil in ghee or milk fat using and it is effective in identifying palm oil adulteration at or above 5%. So, this method could be adopted by any regulatory body to ascertain the quality of ghee.

**Table S1 Equation for calculate the S-value of pure ghee and adulterated ghee samples.**

| **S-value** | **Equation employed** |
| --- | --- |
| S1 | = (2.0983C30) + (0.728C34) + (0.6927C36) + (0.6353C38) + (3.7452C40) ‒ (1.2929C42) + 1.3544C44) + (1.7013C46) + (2.5283C50) |
| S2 | = (3.7453C32) + (1.1134C36) + (1.3648C38) + (2.1544C42) + (0.4273C44) + (0.5809C46) + (1.2926C48) + (1.0306C50) + (0.9953C52) + (1.2396C54) |
| S3 | = (3.6644C28) + (5.2297C30) ‒ (12.5073C32) + (4.4285C34) ‒ (0.2010C36) + (1.2791C38) + (6.7433C40 – (4.2714C42 + (6.3739C46) |
| S4 | = (6.5125C26) + (1.2052C32) + (1.7336C34) + (1.7557C36) + (2.2325C42) + (2.8006C46) + (2.5432C52) + (0.9892C54) |
| S total | = (2.7575C26) + (6.4077C28) + (5.5437C30) ‒ (15.3247C32) + (6.2600C34) + (8.0108C40) ‒ (5.336C42) + (0.6356C44) + (6.0171C46) |
| C26 to C54 represents percentage of each major triglycerides fraction containing respective number of carbon atoms in total triglycerides | |

**Table S2 Specification on limits of S-values for pure ghee and adulterated ghee samples.**

| **S-value** | **Equations** | **Limits** |
| --- | --- | --- |
| S1 | Sunflower; Soya bean; Rape seed; olive; Linseed; maize germ; Wheat germ; Cotton seed & Fish oil | 98.05 - 101.95 |
| S2 | Coconut & Palm kernel fat | 99.42 - 100.58 |
| S3 | Palm oil & beef tallow detection | 95.90 - 104.10 |
| S4 | Lard | 97.96 - 102.04 |
| S total | Total general formula for any foreign fat | 95.68 - 104.32 |
